# Single-Stage PET Waste Upcycling into Copolymers: Study of Thermal Properties and Crystallization

**DOI:** 10.3390/polym18202587

**Published:** 2026-10-20

**Authors:** Kirill A. Kirshanov, Gadir Sh. Aliev, Daniil A. Ismaylov, Nadezhda A. Lobanova, Anton M. Pogorely, Roman V. Toms, Alexander Yu. Gervald

**Affiliations:** M.V. Lomonosov Institute of Fine Chemical Technologies, MIREA—Russian Technological University, Moscow 119571, Russia; zaligad99@gmail.com (G.S.A.); ismaylov.daniil@bk.ru (D.A.I.); lavanda20002000@yandex.ru (N.A.L.); pogorely.mitht@yandex.ru (A.M.P.); gervald@bk.ru (A.Y.G.)

**Keywords:** polyethylene terephthalate, PET, polyester, polymer waste, chemical recycling, upcycling, transesterification, copolymers, thermal properties, crystallization

## Abstract

The transesterification of polyester with hydroxyl-terminated oligoesters represents a promising route for the chemical upcycling of polyethylene terephthalate waste. The single-stage melt process under investigation yielded value-added PET-based copolyesters containing up to 15 mol.% diethylene glycol and triethylene glycol units. The effect of comonomer content on the thermal properties and crystallization of the copolymers was studied. It was found that an increase in the number of oxyethylene groups in the comonomer leads to a greater decrease in the glass transition temperature and the isothermal crystallization rate. Diethylene glycol was shown to lower the equilibrium melting temperature more than triethylene glycol. Thus, during PET upcycling, the glass transition temperature decreased from 80 to 50 °C, and the melting temperature from 280 to 250 °C.

## 1. Introduction

One of the key environmental issues of our time is the anthropogenic impact on the environment [1]. Within this context, plastic waste management is one of the most pressing challenges facing modern industry. Initiatives to reduce polymer production and consumption do not address the problem posed by existing plastic waste. Therefore, recycling remains the primary focus of waste management for the benefit of a circular economy.

Recycling is classified as mechanical, chemical, and incineration. Although mechanical recycling is currently the most cost-effective and widespread, repeated mechanical recycling results in deterioration of material properties. Incineration is an important method of polymer disposal at the end of its life cycle; however, it contributes to the emission of carbon dioxide and the release of various combustion products.

Chemical recycling offers the most promise [2,3]. It includes pyrolysis, gasification, and hydrogenation of waste to produce hydrocarbon mixtures, as well as various solvolysis technologies for producing fine organic compounds. Solvolysis technologies are known to be suitable only for polymers with reactive functional groups in the main chain.

The largest class of such polymers by production volume is polyesters—primarily polyethylene terephthalate (PET) [4,5,6,7,8]. The main reason for the low recovery rate of PET waste is the limited range of applications for recycled products.

Moreover, the main product of PET waste recycling is currently recycled PET pellets, which are significantly inferior to virgin PET in terms of processing and functional properties. Furthermore, many commercial forms of PET, including post-consumer polyester fibers [9], polyester tire cord waste [10], and multilayer or metallized films [11], cannot be mechanically recycled.

The main products of chemical recycling of polyethylene terephthalate are monomers that can be used to synthesize PET with properties comparable to those of virgin PET, such as bis(2-hydroxyethyl) terephthalate, dimethyl terephthalate, and terephthalic acid, which can be obtained by various glycolysis [12,13,14,15,16], alcoholysis [17,18,19,20,21], and hydrolysis [22,23,24,25,26] methods.

Another approach is to convert PET waste into value-added polyester products through a process known as upcycling. These products include plasticizers, dyes, Schiff bases [27,28], polyester resins [29,30], and copolyesters designed for various applications [31].

The most studied method for producing copolyesters from PET waste is depolymerization followed by repolymerization [31,32,33]. Another approach involves interchain exchange between polyesters via transesterification [34,35,36,37,38,39]. Previous studies have investigated this approach, primarily for polyethylene terephthalate/polyethylene naphthalate blends [34,35], but these processes have not been adopted industrially due to the low reaction rates.

In 2022, we proposed using oligoesters as glycolysis agents for the production of phthalate-containing copolyesters [40] and unsaturated polyester resins [41]. We noted that oligoesters do not evaporate at the melting point of polyethylene terephthalate and have a sufficiently high concentration of reactive hydroxyl groups for rapid transesterification. The novelty of this approach lies in conducting a homogeneous reaction in the melt, which is not feasible without elevated pressure for most conventional glycolysis agents. In 2023, a process using an antibacterial monomer prepolymerized with ethylene glycol was described by Zhang et al. [42].

In 2025, we described the kinetics of transesterification reactions between polyethylene terephthalate and polyesters with terminal hydroxyl groups [43]. We showed that the process involves the simultaneous occurrence of numerous transesterification reactions—between the hydroxyl and ester groups of the terminal and bound units of polyethylene terephthalate and the oligoester—as well as glycolysis and ester exchange. These reactions proceed via a well-established transesterification mechanism. The proposed kinetic model allows one to predict the molecular weight characteristics and degree of randomness of the resulting copolymer from the concentrations of the reactants, the pre-exponential factor of the Arrhenius equation, the activation energy, the reaction temperature, and the composition of the recycling agent.

The primary goal of producing PET-based copolymers is to lower the glass transition and melting temperatures and reduce the crystallization rate. A reduced crystallization rate enables the production of transparent parts or parts with lower shrinkage without the high costs of rapid cooling [44]. Thermal properties, such as glass transition and melting temperatures, influence the processing window and service-temperature range of the polymer. This is especially important for the rapidly developing field of FDM/FFF 3D printing, where PET-G is one of the main polymers used. The main comonomers used are orthophthalate [40] and isophthalate [45], trimethylene glycol [46,47], butylene glycol [48], neopentyl glycol [44], and 1,4-cyclohexanedimethanol [49,50].

The thermal properties and crystallization behavior of copolymers containing diethylene glycol (DEG) units have been studied in the past [51,52], due to the formation of diethylene glycol during the synthesis and recycling of polyethylene terephthalate via an etherification side reaction. The properties of copolymers containing triethylene glycol (TEG) units have not yet been investigated. Moreover, the relevance of these monomers for modifying polyethylene terephthalate has increased in recent years not only due to their byproduct formation, but also due to their use in chemical recycling and upcycling [53,54,55].

Therefore, the aim of this study is to investigate the thermal properties and crystallization of PET-based copolymers containing diethylene glycol and triethylene glycol units, prepared for the first time via single-stage chemical upcycling of polyethylene terephthalate waste.

## 2. Materials and Methods

### 2.1. Materials

Polyethylene terephthalate (PET) with a number-average molecular weight of 30,000 g mol^−1^ was used in the commercial form of flakes [43].

The other reagents were purchased from Sigma-Aldrich (St. Louis, MO, USA). Tetrahydrofuran (THF, 99%) used for gel permeation chromatography was distilled at atmospheric pressure before use. Boiling point T_b_(THF) = 66 °C, refractive index n_D_^20^(THF) = 1.407.

The glycol monomers diethylene glycol (DEG, 99%) and triethylene glycol (TEG, 99%) were purified by vacuum distillation. T_b_(DEG) = 245 °C, n_D_^20^(DEG) = 1.447; T_b_(TEG) = 285 °C, n_D_^20^(TEG) = 1.456.

Dimethyl terephthalate (DMT, 99%) and the transesterification catalyst antimony (III) oxide (Sb_2_O_3_, 99%) were used without further purification.

### 2.2. Synthesis of Oligoesters with Terminal Hydroxyl Groups

Oligodiethylene terephthalate (ODT) and oligotriethylene terephthalate (OTT) with terminal hydroxyl groups were obtained by melt polycondensation. The monomers diethylene glycol and dimethyl terephthalate for ODT and triethylene glycol and dimethyl terephthalate for OTT were taken in a molar ratio of 5:4. Antimony trioxide catalyst was used in an amount of 0.1 wt.% relative to the mass of dimethyl terephthalate.

The stirred-tank reactor was charged with the reaction mixture components. The reactor was maintained at 140 °C for 90 min under an inert gas flow and a stirring speed of 100 rpm. The temperature was then increased by 10 °C and the stirring speed by 50 rpm every 30 min. Once the mixture reached 190 °C and 350 rpm, a vacuum line was connected and the process was continued until methanol evolution ceased.

### 2.3. PET Upcycling into Copolymers

To obtain the copolyester, polyethylene terephthalate and the synthesized oligoester with terminal hydroxyl groups were melt-mixed at the molar ratios of repeating units given in Table 1.

Polyethylene terephthalate and oligoester (ODT, OTT) were melted at 280 °C with stirring at 50 rpm under an inert gas. The reaction mixture was then held for 90 min under the same conditions. Residual antimony trioxide from previously synthesized ODT and OTT oligoesters served as the transesterification catalyst.

### 2.4. Characterization of PET, Oligoesters and Transesterification Products

#### 2.4.1. Fourier Transform Infrared (FTIR) Spectroscopy

The composition of the synthesized OTT and ODT oligoesters was confirmed by the position of the characteristic absorption bands in the FTIR spectra. The spectra were recorded using a Spectrum 65 FT-IR spectrometer (Perkin Elmer, Waltham, MA, USA) and processed using Spectrum v. 10.6 software. The characteristic absorption bands required for the identification of the oligoesters [56] are listed in Table 2.

#### 2.4.2. Gel Permeation Chromatography (GPC)

The molecular weight distribution of the synthesized OTT and ODT oligoesters and the resulting PDET-95:5, PDET-90:10, PDET-85:15, PTET-95:5, PTET-90:10, and PTET-85:15 copolyesters was determined by gel permeation chromatography using a Gilson Inc. (Middleton, WI, USA) instrument equipped with an Agilent MIXED-E column (Santa Clara, CA, USA).

For sample preparation, the oligomer or copolymer was dissolved in tetrahydrofuran at a concentration of 1.0 mg mL^−1^. Polystyrene standards from Agilent (Santa Clara, CA, USA) with peak molecular weights of 580, 1280, 2940, 10,110, and 28,770 g mol^−1^ and a narrow molecular weight distribution of no more than 1.12 were used. The results were corrected using the constants of the Kuhn–Houwink equation. Measurements were performed at a flow rate of 1 mL min^−1^ at a temperature of 25 °C.

#### 2.4.3. Oligoester Synthesis Conversion

The conversion of OTT and ODT synthesis was determined based on the ratio of monomers with different functional groups and the average molecular weight determined by gel permeation chromatography.

Conversion α, %, was calculated using Equation (1).

(1) $$P=\frac{1+q}{1+q\times (1-2\alpha )},$$

where P is the degree of polycondensation, and q is the stoichiometric imbalance factor.

The degree of polycondensation was determined using Equation (2).

(2) $$P=\frac{M_{n}}{{MM}_{av}},$$

where M_n_ is the number-average molecular weight, g mol^−1^, and MM_av_ is the average molecular weight of the unit, g mol^−1^.

The stoichiometric imbalance factor was determined using Equation (3).

(3) $$q=\frac{N_{X0}}{N_{Y0}},$$

where N_X0_ and N_Y0_ are the amounts of glycols and dicarboxylic acids or their derivatives in the initial mixture of monomers, N_X0_ ≥ N_Y0_.

#### 2.4.4. Differential Scanning Calorimetry (DSC)

Calorimetric studies were performed on a DSC 204 F1 Phoenix calorimeter (NETZSCH Geratebau GmbH, Germany) in the heating mode from 50 to 300 °C. DSC curves were obtained in an inert atmosphere (argon) at a scanning rate of 10 deg min^−1^.

### 2.5. Experimental Data Analysis

#### 2.5.1. Glass Transition Temperature Models

The glass transition temperatures of the copolyesters PDET-95:5, PDET-90:10, PDET-85:15, PTET-95:5, PTET-90:10 and PTET-85:15 were determined from the DSC curves. The obtained experimental data were processed using the Fox, Gordon–Taylor, Kwei, and Brostow models according to Equations (4)–(7).

(4) $$\frac{1}{T_{g}}=\frac{x_{1}}{T_{g1}}+\frac{1-x_{1}}{T_{g2}},$$

where T_g_ is the glass transition temperature of the copolymer, K; T_g1_, T_g2_ are the glass transition temperatures of homopolymers, K; and x_1_ is the proportion of units with the glass transition temperature of the homopolymer T_g1_.

(5) $$T_{g}=\frac{x_{1}\times T_{g1}+k_{GT}\times (1-x_{1})\times T_{g2}}{x_{1}+k_{GT}\times (1-x_{1})},$$

where k_GT_ is the Gordon–Taylor entropy constant.

(6) $$T_{g}=\frac{x_{1}\times T_{g1}+k_{Kw}\times (1-x_{1})\times T_{g2}}{x_{1}+k_{Kw}\times (1-x_{1})}+q\times x_{1}\times (1-x_{1}),$$

where k_Kw_ is the Kwei entropy constant.

(7) $$T_{g}=x_{1}\times T_{g1}+\left(1-x_{1}\right)\times T_{g2}+x_{1}\times \left(1-x_{1}\right)\times \;\left[a_{0}+a_{1}\times \left(2x_{1}-1\right)z+a_{2}\times {\left(2x_{1}-1\right)}^{2}+a_{3}\times {\left(2x_{1}-1\right)}^{3}\right],$$

where a_0_, a_1_, a_2_, and a_3_ are Brostow approximation constants.

#### 2.5.2. Melting Temperature Models and Hoffman–Weeks Plot

Equilibrium melting temperatures were determined using Hoffman–Weeks plots. The plots show the dependence of the melting temperature on the isothermal crystallization temperature [57].

Based on published data for polyethylene terephthalate, isothermal crystallization temperatures of 170, 180, and 190 °C were selected. The samples were held isothermally for 60 min. To determine the melting points, the sample was heated to 300 °C at a scanning rate of 10 deg min^−1^.

The obtained melting temperatures were processed using the Flory, Baur and Wendling–Suter models according to Equations (8)–(10).

(8) $$\frac{1}{T_{m}^{0}}=\frac{1}{T_{m}}+\frac{R}{H_{m}^{0}}\times ln(x_{1}),$$

where T_m0_ is the equilibrium melting temperature, K; H_m0_ is the equilibrium heat of fusion, J mol^−1^; and R is the universal gas constant, 8.314 J mol^−1^ K^−1^.

(9) $$\frac{1}{T_{m}^{0}}=\frac{1}{T_{m}}+\frac{R}{H_{m}^{0}}\times ln(x_{1}-{\langle \xi \rangle }^{-1}),$$

where 〈ξ〉 is the average length of the homopolymer sequence in the melt.

(10) $$\frac{1}{T_{m}^{0}}=\frac{1}{T_{m}}+\frac{R}{H_{m}^{0}}\times ln(x_{1}+(1-x_{1})\times e^{-\epsilon /RT}-{\langle \xi \rangle }^{-1}),$$

where ϵ is the excess free energy of the defect created by the introduction of one comonomer unit into the crystal, J mol^−1^.

#### 2.5.3. Avrami Plot

Based on the obtained crystallization isotherms in Avrami coordinates [57], the rate constants and exponents of the Avrami equation were determined for the copolyesters PDET-95:5, PDET-90:10, PDET-85:15, PTET-95:5, PTET-90:10, and PTET-85:15 using Equation (11).

(11) $$X_{t}=(1-e^{{-(k}_{a}\times {t)}^{n_{a}}}),$$

where X_t_ is the relative crystallinity at time t, k_a_ is the Avrami rate constant, and n_a_ is the Avrami exponent.

The crystallization half-times t_1/2_, s, for the copolyesters PDET-95:5, PDET-90:10, PDET-85:15, PTET-95:5, PTET-90:10, and PTET-85:15 were determined using Equation (12).

(12) $$t_{1/2}=60\times {\left(\frac{ln(2)}{10^{k_{a}}}\right)}^{\frac{1}{n_{a}}},$$

#### 2.5.4. Hoffman–Lauritzen Theory

To describe the dependence of isothermal crystallization half-times on isothermal crystallization temperature, the Hoffman–Lauritzen theory was used. For this purpose, the pre-exponential factor and activation energy of the Hoffman–Lauritzen equation were determined from the Hoffman–Lauritzen coordinate plots [58] for copolymers of different compositions using Equations (13)–(15).

(13) $$\frac{1}{t_{1/2}}=A\times e^{\left(\frac{-U^{*}}{R\times (T_{c}-T_{\infty })}\right)}\times e^{\left(\frac{-K_{g}}{T_{c}\times (T_{m}^{0}-T_{c})\times f}\right)},$$

where A is the pre-exponential factor; U* is the activation energy of the transfer of polymer chains to the crystal-melt boundary, 6.3 kJ mol^−1^ for most polymers; K_g_ is the activation energy of the formation of a crystalline nucleus of critical size, K^2^; T_c_ is the temperature of isothermal crystallization, K; T_∞_ is the temperature at which chain mobility ceases (infinite viscosity), K; and f is the temperature correction factor.

(14) $$T_{\infty }=T_{g}-30,$$

(15) $$f=\frac{2T_{c}}{T_{m0}+T_{c}},$$

## 3. Results and Discussion

### 3.1. Oligoesters and Copolymers Characterization

Initially, oligoesters with terminal hydroxyl groups were obtained and characterized. The appearance of the synthesized oligoesters is shown in Figure 1.

FTIR spectra of ODT and OTT are shown in Figure 2.

The spectra of ODT and OTT samples exhibit prominent bands at 1270 and 939 cm^−1^, corresponding to the ether group. The oligomers exhibit characteristic absorption bands at 1116 and 1176 cm^−1^ (terephthalate), 1715 cm^−1^ (carbonyl group, stretching), and 1243 cm^−1^ (ester group, stretching). The presence of an absorption band at 3350 cm^−1^ (hydroxyl group, stretching) confirms that ODT and OTT oligoesters have terminal hydroxyl groups.

The number-average and weight-average molecular weights, polydispersity index, and conversion calculated from the results of gel permeation chromatography are given in Table 3.

By reacting polyethylene terephthalate with oligodiethylene terephthalate and oligotriethylene terephthalate, we obtained poly(diethylene-co-ethylene terephthalates) PDET-95:5, PDET-90:10, and PDET-85:15 and poly(triethylene-co-ethylene terephthalates) PTET-95:5, PTET-90:10, and PTET-85:15.

The maximum mole fraction of the glycol comonomer was set at 15%, since with a further increase in the content of di- and triethylene glycol the copolymers become completely amorphous, precluding the analysis of melting and crystallization behavior.

Polyester fibers were drawn from the melt; the appearance of the obtained copolymers is shown in Figure 3. The degrees of randomness calculated using the kinetic model [43] and NMR spectra, as well as the number-average molecular weights, are given in Table 4.

Apparently, the decrease in molecular weight with increasing diethylene glycol and triethylene glycol content is associated with a higher proportion of low-molecular-weight hydroxyl-terminated oligoethers (ODT or OTT) in the reaction mixture. The resulting polyester products were studied using differential scanning calorimetry. The DSC curves obtained during heating for amorphous copolymer samples obtained by rapid melt cooling are shown in Figure 4.

Increasing the glycol comonomer content to 15% reduces the observed melting temperatures and heats of fusion. Adding diethylene glycol reduces the melting temperature from 250 to 220 °C, and the heat of fusion from 71 to 40 J g^−1^.

Adding triethylene glycol reduces the melting temperature to 214 °C, and the heat of fusion to 29 J g^−1^. The reduction in melting temperature and heat of fusion confirms the disruption of the crystal structure due to changes in chain regularity relative to polyethylene terephthalate, which is consistent with theoretical concepts.

### 3.2. Glass Transition Temperatures

Figure 5 shows the dependence of glass transition temperatures on the content of ethylene glycol units, processed using various mathematical models.

The Fox model for ideal mixtures shows the poorest agreement with experimental data, as it assumes the simple exclusion of the comonomer from the mixture. The Gordon–Taylor model shows better agreement. The complex approximation models of Kwei and Brostow, which account for strong intermolecular interactions (such as hydrogen bonding between hydroxyl and ester groups), show the best agreement.

With the same increase in the content of diethylene glycol units, a decrease in the glass transition temperature can be observed from 78 to 62 °C, and with an increase in triethylene glycol content, it decreases to 50 °C. Triethylene glycol has a stronger effect because it disrupts the regularity of polymer chain packing to a greater extent, as evidenced by a greater decrease in melting point. Triethylene glycol is also a long, linear ether and increases chain flexibility more than diethylene glycol.

### 3.3. Equilibrium Melting Temperatures (Hoffman–Weeks)

To determine the equilibrium melting temperatures, DSC curves were obtained for the copolyesters PDET-95:5, PDET-90:10, PDET-85:15, PTET-95:5, PTET-90:10, and PTET-85:15 subjected to isothermal crystallization at temperatures of 170, 180, and 190 °C. An example of a DSC curve is shown in Figure 6; all curves are shown in Figure S1 in the Supplementary Materials.

Peaks I, II, and III (Figure 6) are attributed to the melting of lamellae formed during secondary crystallization, the melting of lamellae formed during primary crystallization, and recrystallization, respectively. To determine the equilibrium melting temperature using the Hoffman–Weeks plot, it is assumed that the enthalpy of melting of peak II (Figure 6) is due only to the melting of crystals formed during isothermal crystallization. In this case, the corresponding temperatures of peak II (Figure 6) are used as the melting temperature. Figure 7 shows the Hoffman–Weeks plots from which the equilibrium melting temperatures were determined. The values for polyethylene terephthalate are consistent with the literature data [57].

With an increase in comonomer content to 15%, diethylene glycol has the greatest effect on the equilibrium melting point, reducing it from 280 to 249 °C. A similar increase in triethylene glycol concentration has a slightly smaller effect, lowering the equilibrium melting point to 251 °C.

On the one hand, the triethylene glycol unit disrupts the structure of the polymer chain more strongly than the diethylene glycol unit. On the other hand, due to the high conformational mobility of the triethylene glycol unit, which contains three oxyethylene groups, the polymer chain as a whole also exhibits greater conformational mobility than the chain of the diethylene glycol-containing copolymer. This facilitates the mobility of ethylene terephthalate segments within the polymer chain. Thus, we attribute the observed greater reduction in melting temperature caused by diethylene glycol units—compared with that caused by triethylene glycol units—to this factor.

The dependence of the equilibrium melting temperatures of copolyester products on the content of ethylene glycol units is shown in Figure 8.

The change in equilibrium melting temperatures with comonomer content for poly(diethylene-co-ethylene terephthalates) and poly(triethylene-co-ethylene terephthalates) confirms the previously stated hypothesis that diethylene glycol and triethylene glycol units represent defects that disrupt the regularity of the polymer chain and the crystal. The fact that the experimentally obtained values for both PDET and PTET do not agree with the Flory model indicates that they are not simply excluded from the crystal.

### 3.4. Isothermal Crystallization (Avrami)

The primary isothermal crystallization was described using the Avrami equation. The crystallization isotherms of the copolyesters PDET-95:5, PDET-90:10, PDET-85:15, PTET-95:5, PTET-90:10, and PTET-85:15 were obtained at temperatures of 130, 150, 170, 180, and 190 °C. The determined values of the Avrami constants and exponents are presented in Table 5.

The Avrami exponent values at temperatures from 130 to 190 °C for the obtained copolymers PDET-95:5, PDET-90:10, and PDET-85:15 ranged from 2.7 to 2.9, from 2.8 to 3.0, and from 2.7 to 3.0, respectively. For the copolymers PTET-95:5, PTET-90:10 and PTET-85:15, these values ranged from 2.5 to 3.3, from 3.0 to 3.2 and from 2.6 to 3.2, respectively. Since the Avrami exponent values for these copolymers based on polyethylene terephthalate lie mainly in the range from 2.8 to 3.2, it can be assumed that the results are consistent with predominantly three-dimensional growth during isothermal crystallization. Fractional values of the Avrami exponents indicate a combination of mixed nucleation and growth mechanisms.

### 3.5. Isothermal Crystallization (Hoffman–Lauritzen)

Using the measured glass transition temperatures, equilibrium melting temperatures, and crystallization half-times calculated from the results of Avrami analysis, the temperature dependence of isothermal crystallization half-times was described using the Hoffman–Lauritzen theory. This dependence in Hoffman–Lauritzen coordinates is shown in Figure 9.

The pre-exponential factors and activation energies of the Hoffman–Lauritzen equation determined from the equations of the straight lines in Figure 9 are given in Table 6.

The fact that the points lie on two straight lines with different slopes indicates a change in the crystallization regime around 170 °C for copolymers containing 5 and 10% of diethylene glycol or triethylene glycol. For copolyesters containing 15% of comonomer, the point corresponding to isothermal crystallization at 130 °C does not lie on the same straight line as the others, but these data are insufficient to draw a conclusion about the crystallization regime in this case. The resulting relationships between isothermal crystallization half-times and isothermal crystallization temperature in standard coordinates are shown in Figure 10.

## 4. Conclusions

In this work, polyethylene terephthalate-based copolymers containing 5, 10, and 15 mol.% of diethylene glycol and triethylene glycol were investigated.

The copolymers were obtained by reacting polyethylene terephthalate with oligoesters with terminal hydroxyl groups in the melt at 280 °C. Oligodiethylene terephthalate and oligotriethylene terephthalate with a number-average molecular weight ranging from 1250 to 1400 g mol^−1^ were obtained by polycondensation with a conversion of more than 90%. The obtained poly(diethylene-co-ethylene terephthalates) and poly(triethylene-co-ethylene terephthalates) with variable comonomer content have a number-average molecular weight ranging from 7250 to 15 950 g mol^−1^ and a randomness degree from 1.4 to 1.7, which corresponds to a copolymer with an alternation tendency.

For PET-based copolymers containing up to 15 mol.% of diethylene glycol and triethylene glycol, the glass transition temperature decreased from 78 to 62 and 50 °C, respectively, and the equilibrium melting temperature decreased to 249 and 251 °C, respectively.

The Avrami exponent values were found to range from 2.6 to 3.3, indicating three-dimensional crystal growth during isothermal crystallization. The Hoffman–Lauritzen plot indicates a change in the crystallization regime around 170 °C.

Thus, value-added copolymers were obtained by chemical upcycling of polyethylene terephthalate. Reduced crystallinity and slower crystallization increase transparency and reduce shrinkage of the polymer material, simplifying processing. The reduced melting and glass transition temperatures extend the material’s processing window. A potential future application of the products from this single-stage recycling process is 3D printing.

## Figures and Tables

**Figure 1:**
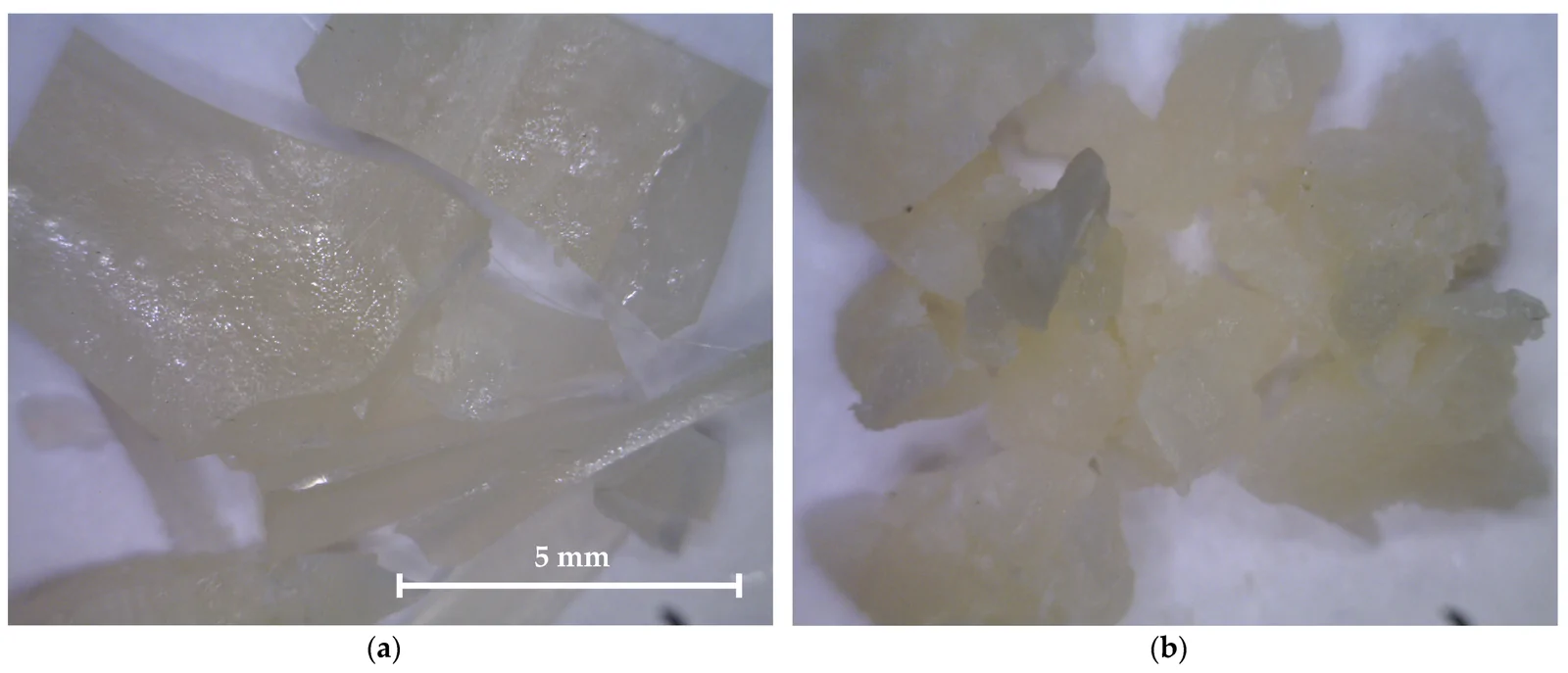
Appearance of oligoesters with terminal hydroxyl groups: (**a**) ODT. (**b**) OTT.

**Figure 2:**
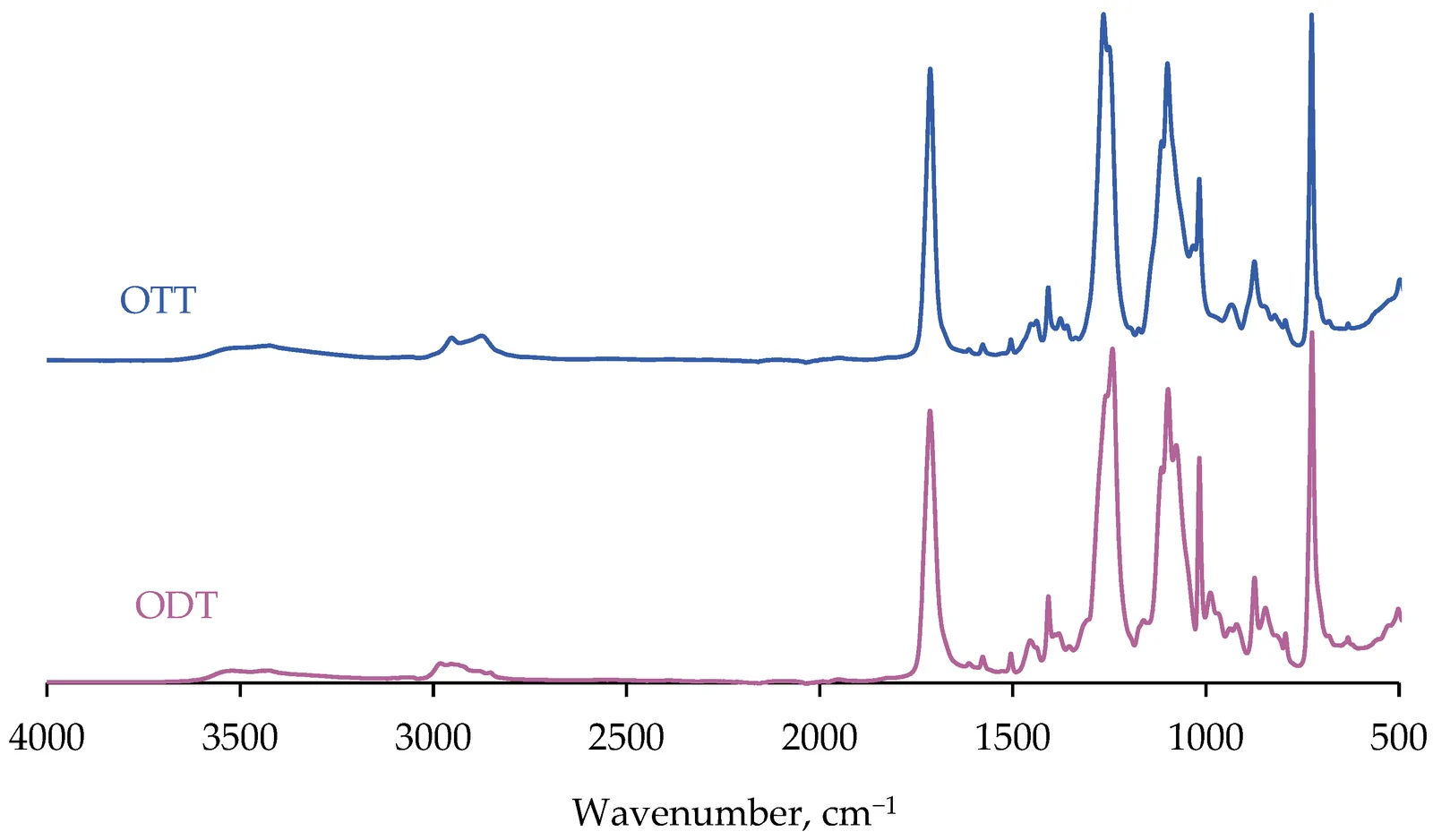
FTIR spectra of ODT and OTT oligoesters with terminal hydroxyl groups.

**Figure 3:**
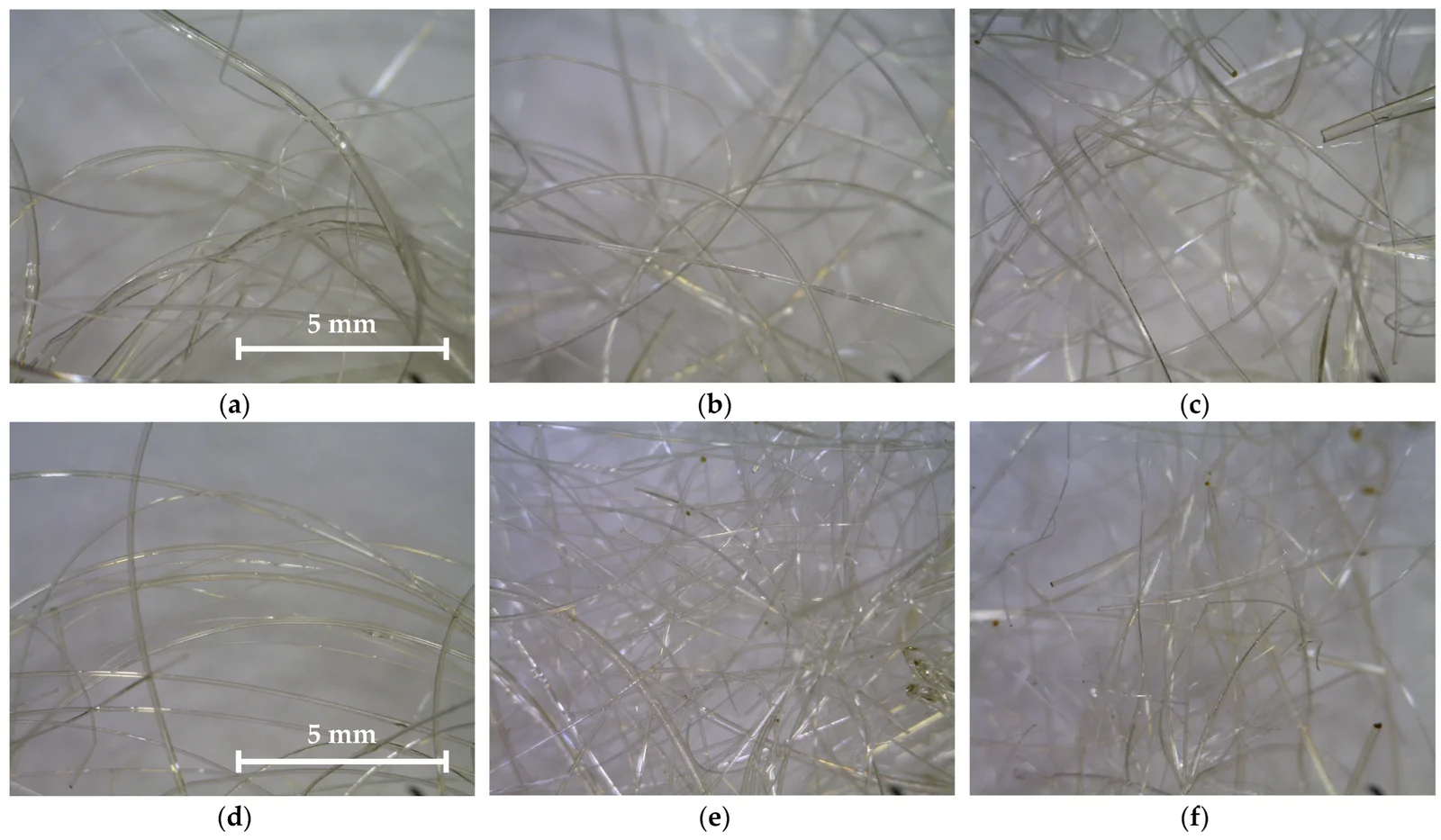
Appearance of copolymers: (**a**) PDET-95:5. (**b**) PDET-90:10. (**c**) PDET-85:15. (**d**) PTET-95:5. (**e**) PTET-90:10. (**f**) PTET-85:15.

**Figure 4:**
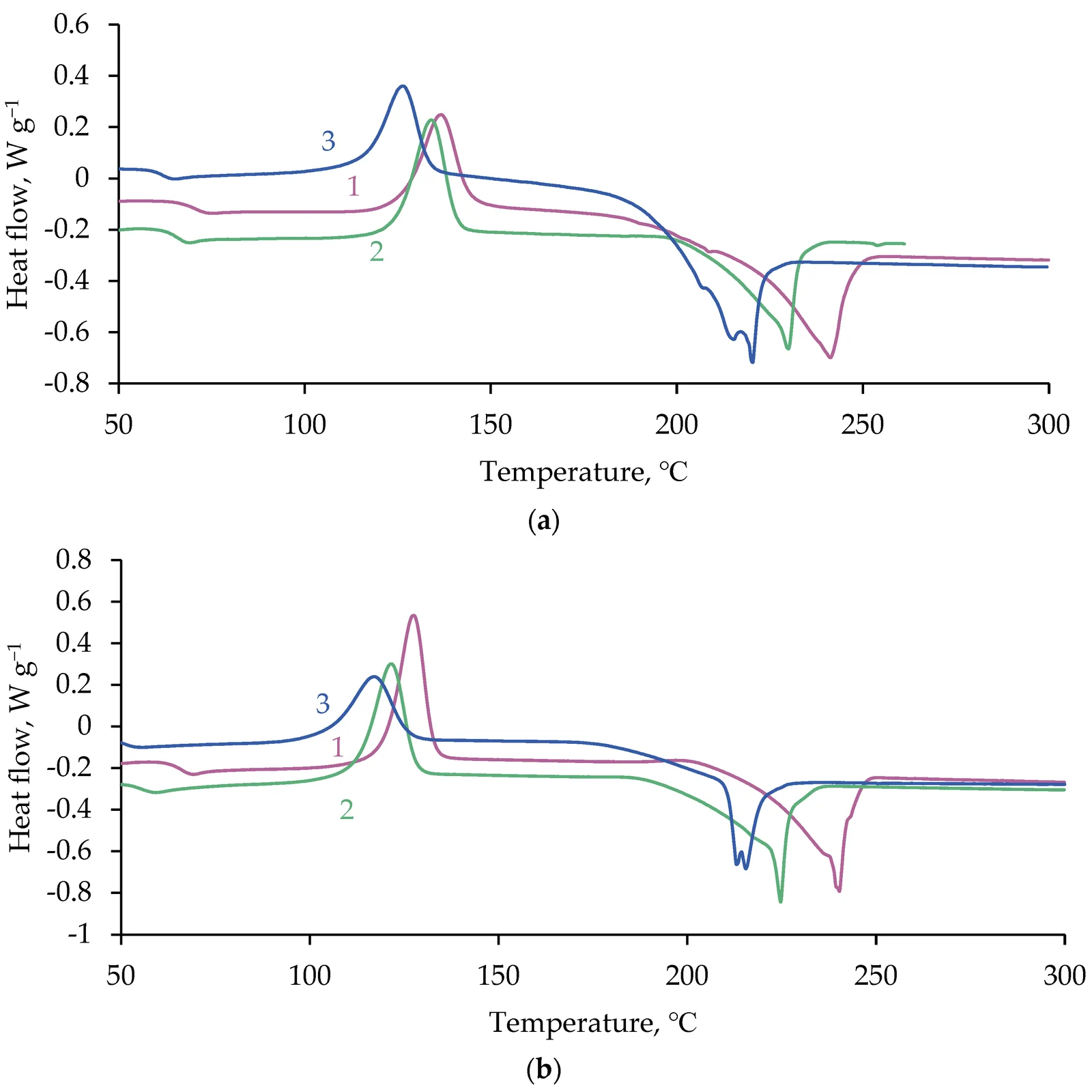
DSC curves during heating for products of interaction of oligoester and PET in ratios of 5:95 (1), 10:90 (2), and 15:85 (3): (**a**) PDET; (**b**) PTET.

**Figure 5:**
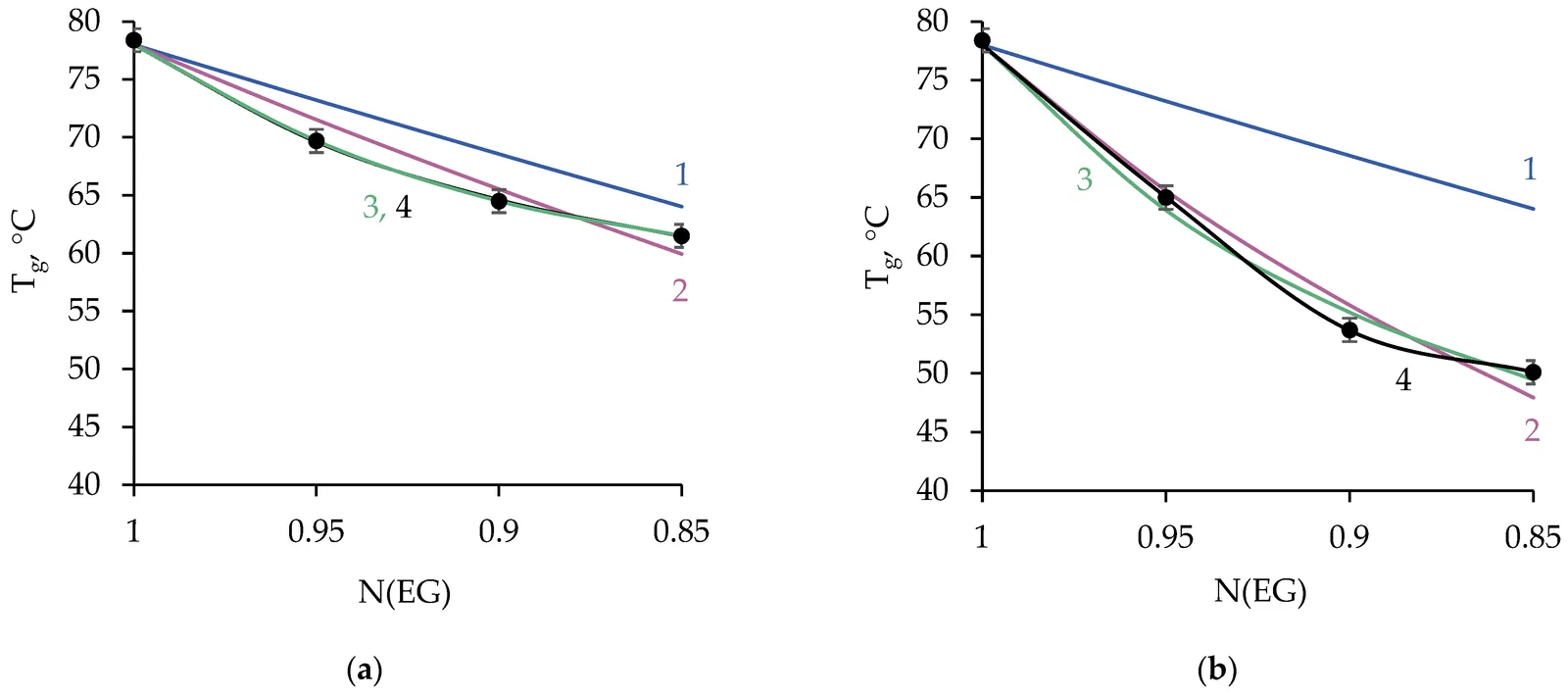
Dependence of glass transition temperatures T_g_ on the content of ethylene glycol N(EG) obtained experimentally (●) and according to the results of calculations using the Fox (1), Gordon–Taylor (2), Kwei (3) and Brostow (4) models for copolymers: (**a**) PDET. (**b**) PTET.

**Figure 6:**
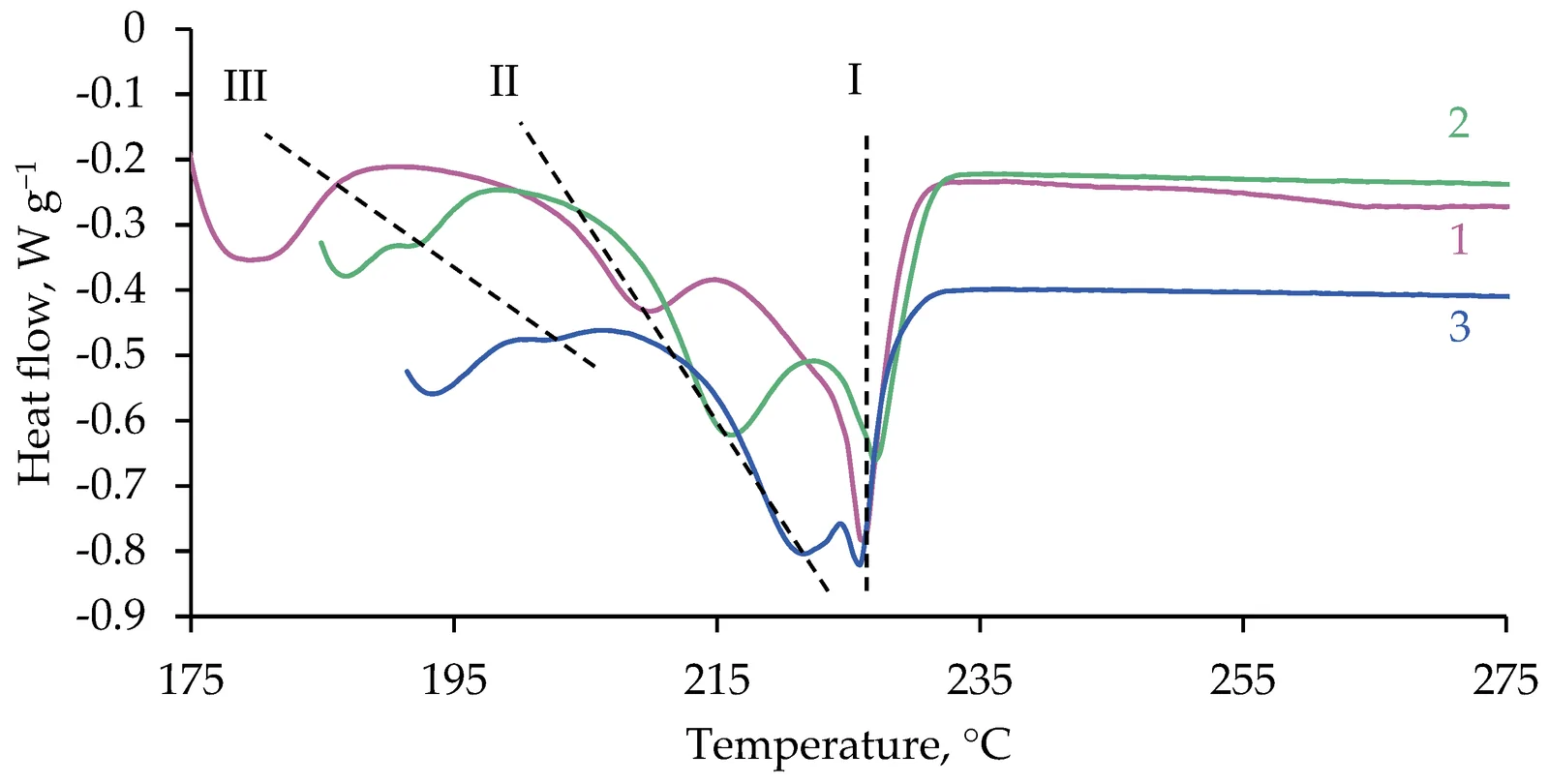
Example of DSC curve at temperatures of 170 (1), 180 (2), and 190 (3) °C.

**Figure 7:**
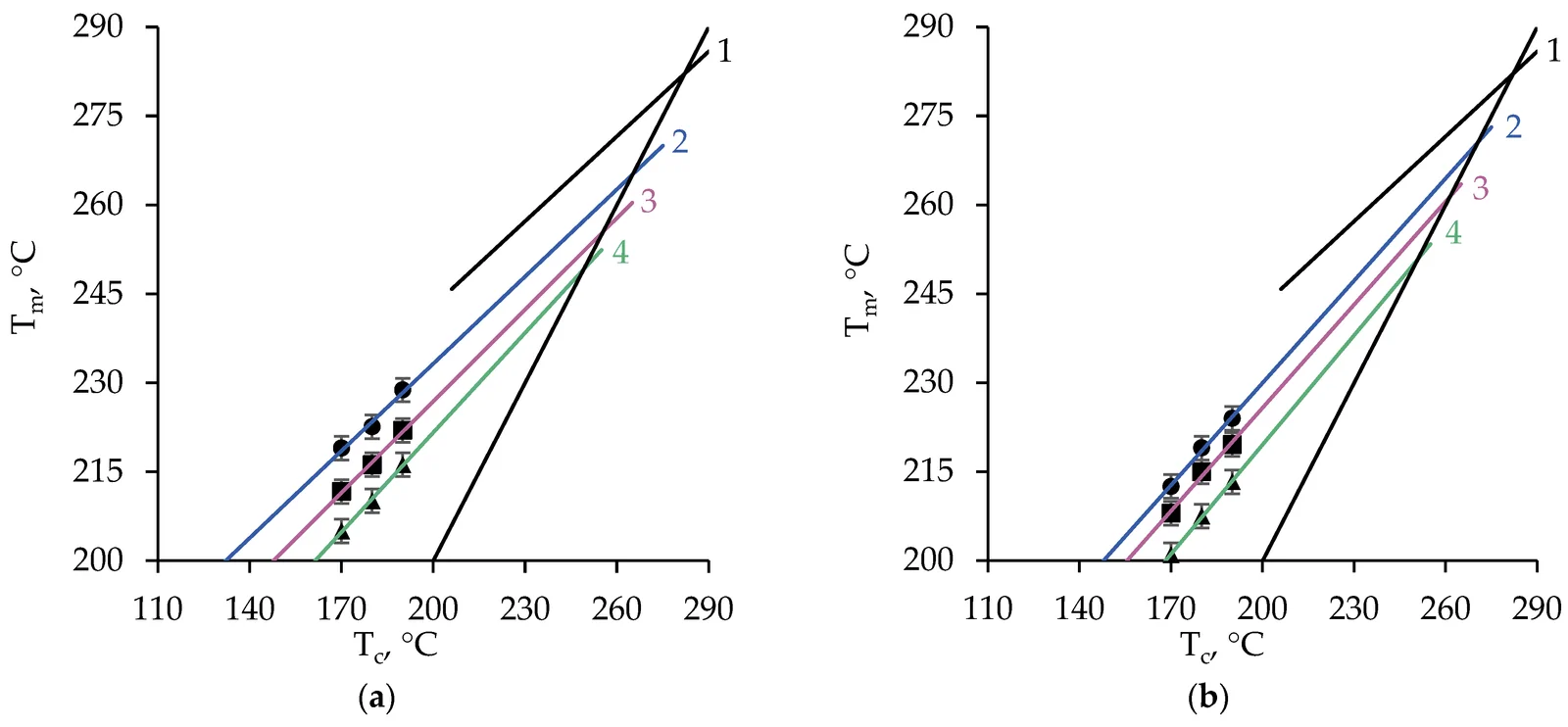
Hoffman–Weeks plots, where T_m_ is the melting temperature, °C; T_c_ is the isothermal crystallization temperature, °C, for PET (1) and PET-based copolymers containing 5 (●, 2), 10 (■, 3), and 15 (▲, 4) mol.% of comonomer, for copolymers: (**a**) PDET. (**b**) PTET.

**Figure 8:**
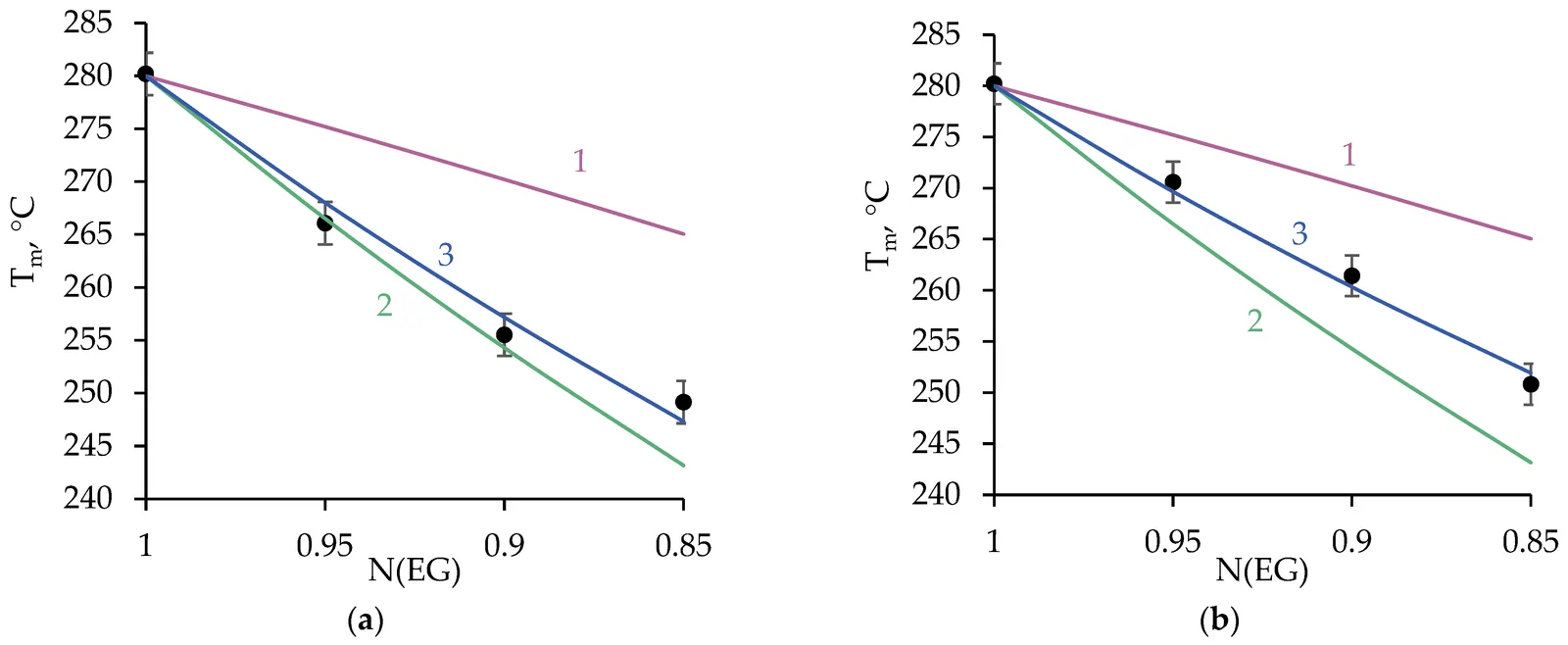
Dependence of the equilibrium melting temperatures T_m_ on the content of ethylene glycol N(EG), obtained experimentally (●) and according to the results of calculations using the Flory (1), Baur (2), and Wendling–Suter (3) models for copolymers: (**a**) PDET. (**b**) PTET.

**Figure 9:**
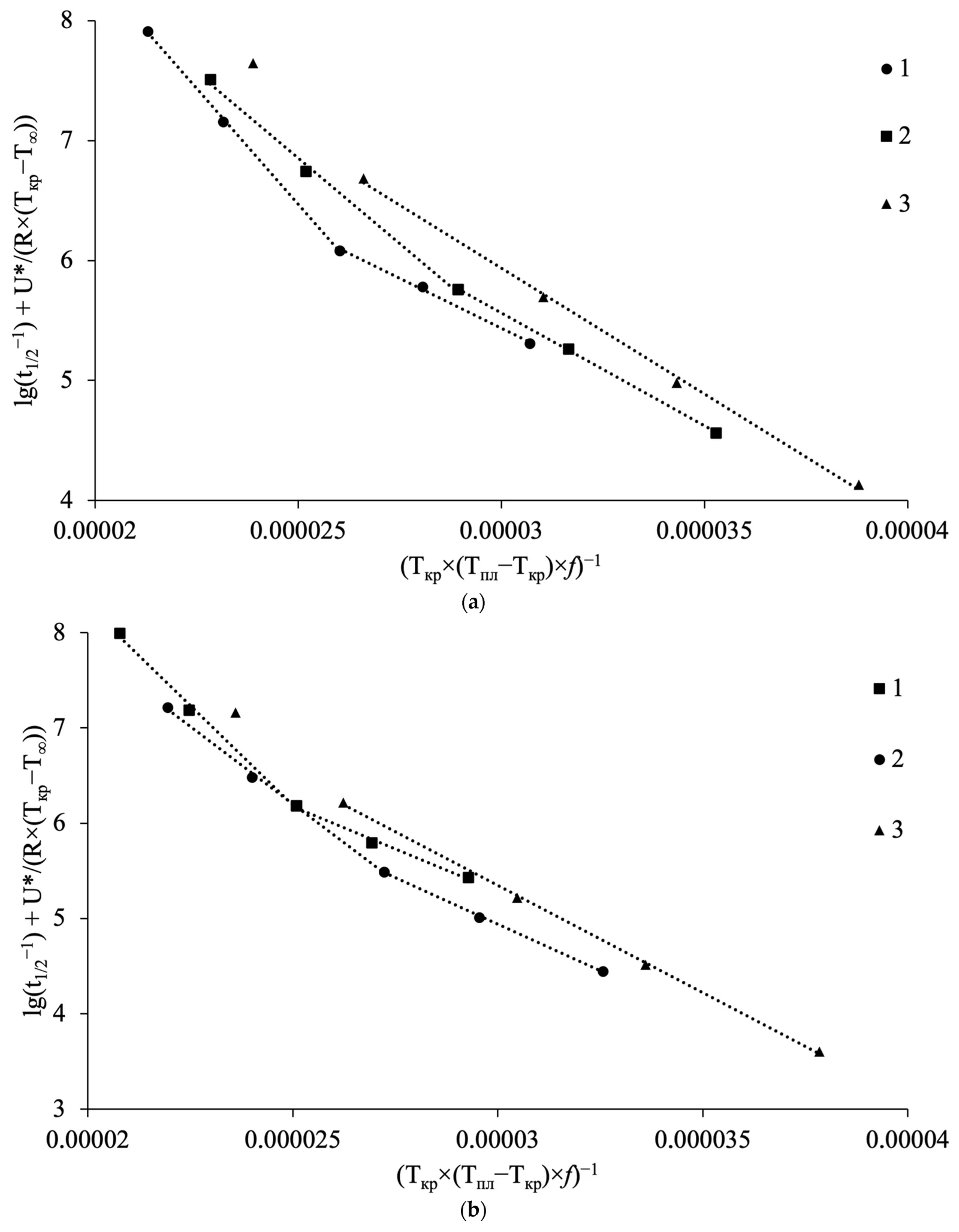
Plots in Hoffman–Lauritzen coordinates for PET-based copolymers containing 5 (1), 10 (2), and 15 (3) mol.% comonomer, for copolymers: (**a**) PDET; (**b**) PTET.

**Figure 10:**
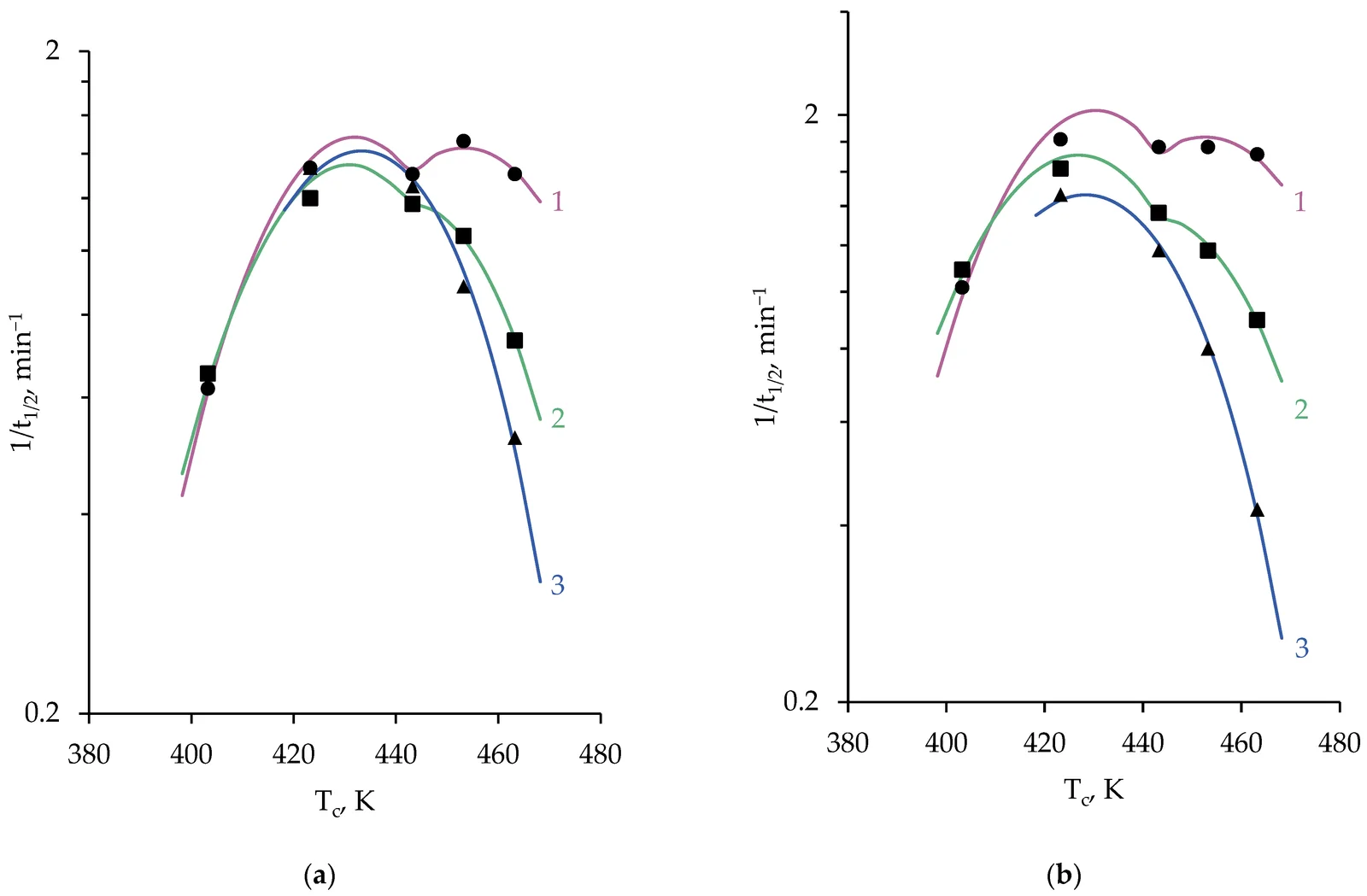
Dependence of the crystallization half-times t_1/2_ on the isothermal crystallization temperature T_c_ for PET-based copolymers containing 5 (●, 1), 10 (■, 2) and 15 (▲, 3) mol.% of comonomer, for copolymers: (**a**) PDET; (**b**) PTET.

**Table 1:** Molar ratios of reagents for the reaction of PET and oligoesters with terminal hydroxyl groups.

Oligoester	Copolymer	PET:Oligoester Molar Ratio
ODT	PDET-95:5	95:5
	PDET-90:10	90:10
	PDET-85:15	85:15
OTT	PTET-95:5	95:5
	PTET-90:10	90:10
	PTET-85:15	85:15

**Table 2:** Characteristic absorption bands of the main groups.

Absorption Band, cm^−1^	Functional Group	Unit
3350	Hydroxyl	Diethylene glycol, triethylene glycol
1270	Ether	
939	Ether	
1715	Carbonyl	Polyester
1243	Ester	
1176	1,4-substituted ring	Terephthalate
1116		

**Table 3:** Characteristics of ODT and OTT samples: number-average (M_n_) and weight-average (M_w_) molecular weight, polydispersity index (PDI) and conversion.

Sample	M_n_, g mol^−1^	M_w_, g mol^−1^	PDI	Conversion, %
ODT	1250	2150	1.7	90.9
OTT	1400	2250	1.6	90.2

**Table 4:** Randomness degrees and number-average molecular weights (M_n_) of the obtained copolymers.

Sample	Randomness Degree	M_n_, g mol^−1^
PDET-95:5	1.7	14,850
PDET-90:10	1.6	9900
PDET-85:15	1.4	7250
PTET-95:5	1.6	15,950
PTET-90:10	1.6	11,100
PTET-85:15	1.5	8450

**Table 5:** Values of the Avrami exponents (n) and constants (K) at different temperatures of isothermal crystallization.

Sample	Temperature, °C	K, min^−n^	n
PDET-95:5	130	0.19	2.7
	150	1.60	2.9
	170	1.42	2.7
	180	2.09	2.9
	190	1.46	2.8
PDET-90:10	130	0.20	2.9
	150	1.18	2.9
	170	1.13	3.0
	180	0.80	2.8
	190	0.28	2.9
PDET-85:15	130	0.60	2.9
	150	1.64	3.0
	170	1.32	2.9
	180	0.49	2.8
	190	0.12	2.7
PTET-95:5	130	0.73	3.3
	150	4.42	3.1
	170	2.87	2.5
	180	3.04	2.6
	190	3.49	3.0
PTET-90:10	130	0.92	3.2
	150	3.26	3.2
	170	1.93	3.3
	180	1.13	3.0
	190	0.50	3.0
PTET-85:15	130	1.62	3.2
	150	2.34	3.2
	170	1.13	3.0
	180	0.37	2.8
	190	0.08	2.6

**Table 6:** Values of pre-exponential factors (ln(${\frac{1}{t_{1/2}}}_{0}$)) and activation energies (K_g_, K^2^) of the Hoffman–Lauritzen equation.

Sample	Temperature, °C	ln(${\frac{1}{t_{1/2}}}_{0}$)	−K_g_, K^2^
PDET-95:5	<170	16.123	−386,197
	>170	10.423	−166,249
PDET-90:10	<170	13.989	−285,316
	>170	11.207	−188,166
PTET-85:15	>150	12.238	−210,011
PTET-95:5	<170	16.635	−417,651
	>170	10.636	−178,402
PTET-90:10	<170	14.330	−325,176
	>170	10.795	−195,139
PTET-85:15	>150	12.090	−224,772

## Data Availability

The original contributions presented in this study are included in the article/Supplementary Material. Further inquiries can be directed to the corresponding authors.

## Supplementary Materials

The following supporting information can be downloaded at https://www.mdpi.com/article/10.3390/polym18202587/s1. Figure S1: DSC curves of copolymers that were subjected to isothermal crystallization pretreatment at 170 (1), 180 (2), and 190 (3) °C: (a) PDET-95:5, (b) PDET-90:10, (c) PDET-85:15, (d) PTET-95:5, (e) PTET-90:10, (f) PTET-85:15.

## Abbreviations

The following abbreviations are used in this manuscript:

PET	Polyethylene terephthalate
ODT	Oligodiethylene terephthalate
OTT	Oligotriethylene terephthalate
ODET	Oligo(diethylene-co-ethylene terephthalate)
OTET	Oligo(triethylene-co-ethylene terephthalate)
FTIR	Fourier transform infrared spectroscopy
DSC	Differential scanning calorimetry
GPC	Gel permeation chromatography
