# Supplementary material for: Single-Stage PET Waste Upcycling into Copolymers: Study of Thermal Properties and Crystallization
Source: Polymers (Basel). 2026 Oct 20;18(20):2587. doi: 10.3390/polym18202587 (PMC13708863; doi:10.3390/polym18202587)
Supplement: Supplementary file 1 [file polymers-18-02587-s001.zip › polymers-4643990-supplementary.pdf]

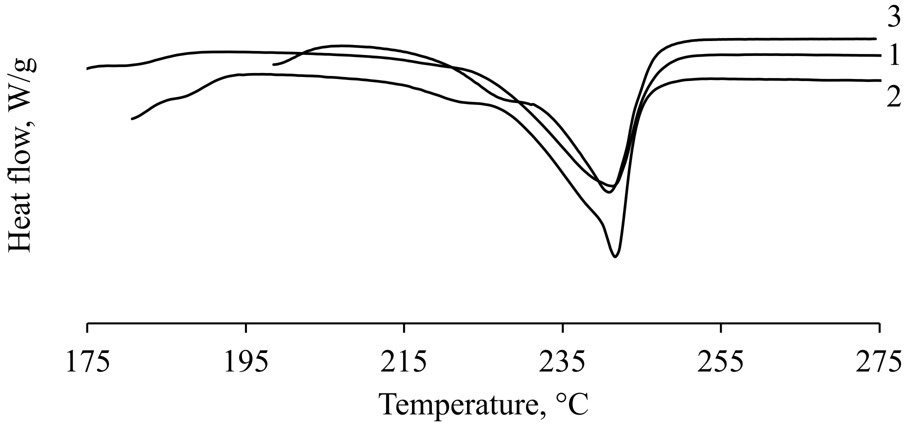
Heat flow, W/g
3
1
2
175
195
215
235
255
275
Temperature, °C
(a)

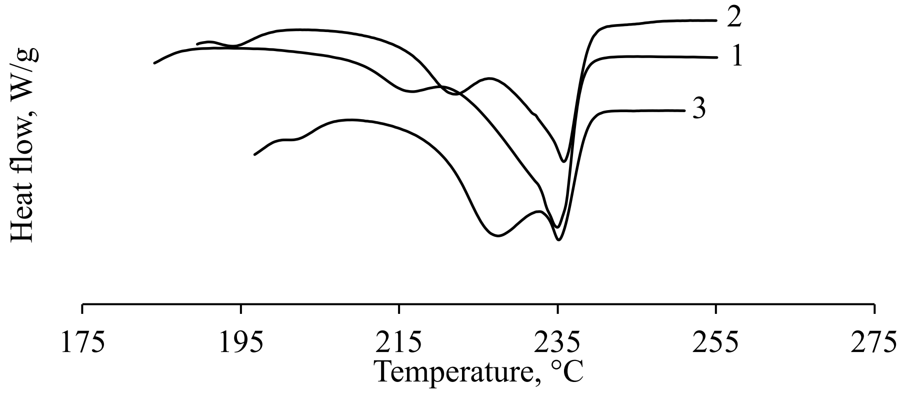
Heat flow, W/g
2
1
3
175
195
215
235
255
275
Temperature, °C
(b)

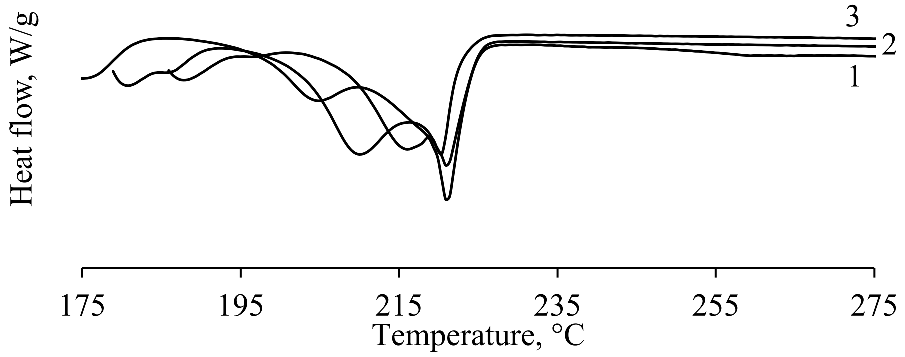
Heat flow, W/g
3
2
1
175
195
215
235
255
275
Temperature, °C
(c)

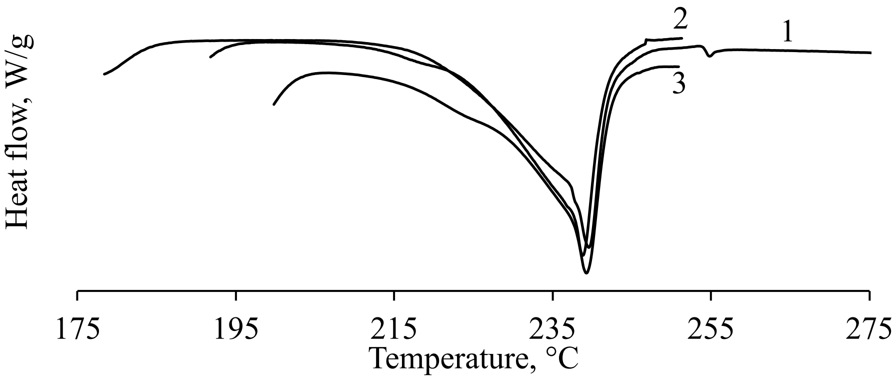

(d)

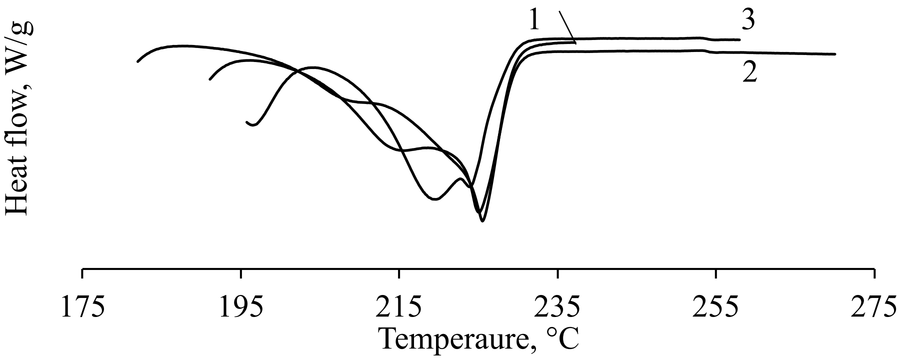

(e)

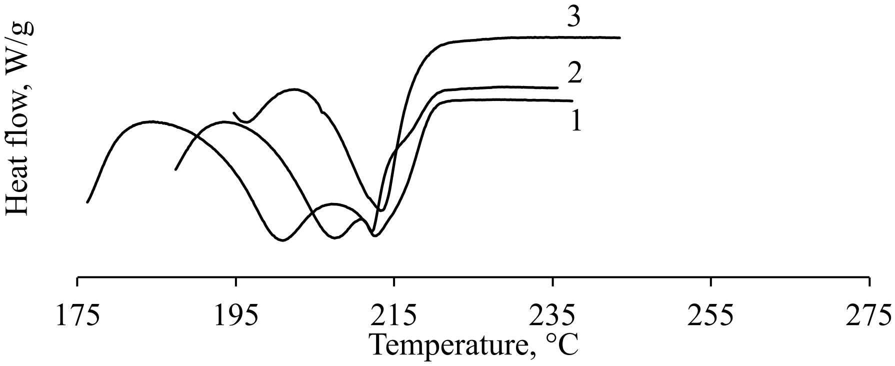

(f)

Figure S1. DSC curves of copolymers that were subjected to isothermal crystallization pretreatment at 170 (1), 180 (2), and 190 (3) °C: (a) PDET-95:5, (b) PDET-90:10, (c) PDET-85:15, (d) PTET-95:5, (e) PTET-90:10, (f) PTET-85:15.
